# Impact of paid work on the academic performance of nursing students

**DOI:** 10.7717/peerj.1838

**Published:** 2016-03-31

**Authors:** Mery Constanza García-Vargas, Mercedes Rizo-Baeza, Ernesto Cortés-Castell

**Affiliations:** 1 Faculty of Nursing, Nursing and Public Health Department, National University of Colombia, Bogotá, Colombia; 2 Faculty of Health Sciences, Nursing Department, University of Alicante, San Vicente del Raspeig, Spain; 3 Pharmacology, Pediatric and Organic Chemistry, University of Miguel Hernández, San Juan de Alicante, Spain

**Keywords:** Academic achievement, Study and work, Nursing

## Abstract

**Background.** Little research exists on the impact of paid work on academic performance of students of health sciences. No research exists on this subject for students in Colombia.

**Objectives.** This paper seeks to analyze the impact of paid work on academic performance among nursing students. Design, settings and participants: cross-sectional research, involving 430 of nursing students from the National University of Colombia (*N* = 566).

**Methods.** Variables analyzed: sex, age, work activity, attendance, current semester, degree subjects studied and unavailable, lost credits, grades during the second semester of 2013, and delayed semesters. Subgroups analyzed: (i) according to labor activity: do not work, work up to 20 h and work more than 20 h per week; (ii) Grade point average: failing is considered as less than 3.0 and passing 3.0 or above out of 5.0. Percentage of delayed semesters were calculated. Qualitative and quantitative variables were analyzed for groups by work activity. The percentage and probability of students getting a grade point average less than 3.0 and delaying semesters were calculated by multivariate logistic regression.

**Results**. A total of 219 of the students work (50.9%), the main reason is socioeconomic, of which 99 (45.2%) work more than 20 h per week and have an increased risk of failing, which is higher in the first semester. They also get lower grades, lose more credits and take longer to finish the degree. The logistic bivariate regressions of success (grade point average, credits gained, courses gained and not having delayed semesters) reduce with work, above all in those who work more than 20 h per week and increase as the number of semesters completed increases, independent of sex.

**Conclusion.** A high percentage of nursing students work more than 20 h per week. The compatibility of paid work with studies in university nursing students has a negative impact on academic performance, more so when they work more than 20 h per week. This negative impact diminishes as the student completes semesters, irrespective of the sex of the students.

## Introduction

The topic of compatibility between paid work and study has drawn a lot of attention for several decades, especially in the USA and Europe, because of the increasing number of working university students, and because of the likely consequences on academic performance and other aspects of college life (Riggert et al., 2006; Lang, 2012; Triventi, 2014). Special analysis is merited for students obtaining a degree in health sciences (medicine, nursing …) because of their hospital internships and contact with patients during their academic formation, which requires considerable eyewitness time to develop their skills, in addition to studying a very demanding program (Moore, 2008).

The compatibility between work and study was studied from the different perspectives (Lang, 2012), always taking into account that the main desire of the university student is to obtain their degree and finish their studies: the time and energy available for the external work (Warren & Cataldi, 2006; Fazio, 2004), the primary orientation of each student for studying or working (Warren, 2002), the primary orientation to work for economic survival (Froment, 2012). But there is a practical consensus that students working more than 16 h per week (equivalent to 2 days a week with an 8 h schedule) experience negative impacts on academic performance (Salamonson & Andrew, 2006; Reyes et al., 2012), because they have less time to study or to do other activities and necessities such as co-curricular activities, social activities, spending time with family or sleeping (Darolia, 2014; Beerkens, Mägi & Lill, 2011; Storey, 2010). Other variables were taken into account in these studies such as age, sex, ethnicity, academic year, social class, workplace and the difficulty in finding a job (Lang, 2012; Riggert et al., 2006; Golden & Baffoe-Bonnie, 2011). Universities have also been studied under this topic, in regards to tuition costs (different among countries and between public and private universities), pedagogical systems, programs and extracurricular activities (or co-curricular events) in order to determine their contributions in the process of learning and socializing (Storey, 2010). A systematic review and meta-analysis show the influences on academic performance from the following factors: personality traits, motivation factors, self-regulatory learning strategies, students’ approach to learning, and psychosocial contextual influences (Richardson, Abraham & Bond, 2012).

Guzmán (2004) found that in the UK and USA, research focused on the characteristics of individuals and work; while in France, articles looked at broader issues such as living conditions when studying, student labor, and other aspects, such as the overcrowding of universities; and in South America, research focuses on socio-economic conditions, labor market participation and the relationship between the education system and the work force.

There is little research on university nursing students with paid work and the impact of work on academic performance. Because of this, there have been studies conducted regarding the impact of work-study on academic performance in England, Ireland, Australia and the USA (Salamonson & Andrew, 2006; Reyes et al., 2012; Rochford, Connolly & Drennan, 2009; Neilson & McNally, 2010; Moore, 2008). Several findings suggest that the impact of work is negative, when work schedules are intense (many working hours). A study in Northern Ireland showed that students working in clinical activities had advantages in clinical practice compared to those who did not work (Hasson, McKenna & Keeney, 2012). Another study complements previous ones and adds that working hours increased as nursing students progress in their studies and in nursing related activities, and reaffirms that working more than 16 h per week is detrimental to academic performance regardless of whether they work in career related activities (Salamonson et al., 2012). In the USA, a study in five public universities showed that social support and stress levels are the factors that affect academic performance in nursing students, rather than working itself (Moore, 2008).

No research has been found in Colombia that addresses the issues facing working university students nor those of working nursing students specifically, which is why this paper seeks to analyze the impact of work on their academic performance, in order to provide knowledge about the controversies raised.

## Material and Methods

### Research question and aims

The research questions raised is: does the number of external working hours affect academic performance in university nursing students? With the following objectives: study the repercussions on certain parameters of academic performance, the simultaneous dedication to a paid job, the intensity of the job, sex and academic year, as well as the following characteristics of work: place, intensity and schedule.

### Study population

The study population is composed of university students pursuing a nursing degree at the National University of Colombia from the first to fifth year (tenth semester) in order to determine the repercussions of work on academic performance.

The program: The duration of the undergraduate program is five years and is organized into 10 semesters (the average duration of study is close to 12 semesters). A total of 161 credits (each credit is equivalent to 48 class and out of class hours) are required and are distributed as follows: 47 credits of fundamentals (Anatomy, Physiology, Biochemistry …), of which the student must pass 10 compulsory and 37 elective courses; 82 credits of Professional Education (Nursing care in health and sickness), of which 66 are compulsory and 16 elective; and 32 elective courses (of any University faculty) which is 20% of the program. A student is forced out or semester to repeat if their weighted grade point average (GPA) is less than 3 out of 5, established by the University. The requirements of the program can be found at: (http://www.legal.unal.edu.co/sisjurun/portal/home.jsp).

### Study design and participants

Cross-sectional observational study applied in 2014 to academic successes of semester II-2013, through a survey structured into 5 parts: personal data (age, sex), conditions of study (class hours, independent study hours, extracurricular activities), academic status (semester enrolled, courses, credits, courses that they couldn’t enroll in), working conditions (paid work, hours/week, place of work, reason for working, work/study preference) and academic performance (GPA of credits gained in the last semester completed, failed courses, lost credits, repeated semesters).

In order to ensure that all the students answering the online survey could understand the questions, it was created with the help of 40 volunteer students separated in two groups and two classrooms. At this time only the survey questions were decided; filling the surveys was done anonymously on a later stage. The application was available online, with institutional permission, during the months of April to August of 2014. All information was recorded in Google and exported Microsoft Office Excel 2007.

Inclusion criteria: Nursing students formally enrolled in the program during the first semester of 2014 (I-2014), except for students starting their first year, for a lack of academic status data. The study includes students who are attending the second to tenth academic semesters. Exclusion criteria: students who opted out of taking the survey and did not give consent.

### Variables analyzed

Variables included were sex, age, hours of work activity, hours of class attendance, hours of independent study, the start date of their studies at the University, the semester in which the student is enrolled, degree courses that students could not register, courses taken, credits gained, credits lost, grade point average in II-2013 period, and the number of delayed semesters, calculated by looking at the difference between the semester that they should be in according to their start date and the semester that they are currently in.

The following subgroups were classifies and formed. Three subgroups were established based on hours worked: 1. those who do not work; 2. those who work up to 20 h per week (half-time work schedule of the full-time work schedule of 40 h/week established by the first objective of the International Labor Organization and official centers in Colombia) and; 3. those working more than 20 h per week. Regarding grade point averages in the last semester studied, less than 3.0 was considered failing, and 3.0 and above was considered passing. Course success was defined as not failing and passing enrolled courses. Credit success was defined as not failing and losing enrolled credits. Similarly, the percentage of delay was calculated using the number of failing semesters in relation to the semester they should be pursuing, defining two subgroups, on track or delayed.

### Statistical study

Qualitative variables were analyzed using absolute and relative frequencies and compared against the sub-working groups using the Chi-square test. For quantitative variables, central tendency (median) and interval (interquartile interval = Quartile 3 − Quartile 1) measures were made, performing the comparison between subgroups using the nonparametric Kruskal–Wallis test as all the variables were shown to have a non-normal distribution. The bivariate logistic regression analysis was used in grades, credits gained, courses and being on track with respect to sex, intensity of paid work, and current semester, calculating the probabilities of different success. The statistical study was done using IBM-SPSS Statistics 23, considering levels of significance of *p* < 0.05.

### Ethical aspects

The research was subjected to the Ethics Committee of the Faculty of Humanities at the National University of Colombia, and was approved on March 27, 2014 (reference number *VIE-013(03/18/2014)*). All students who voluntarily entered the application and answered the survey gave informed consent and were briefed about information confidentiality and anonymity.

**Figure 1:**
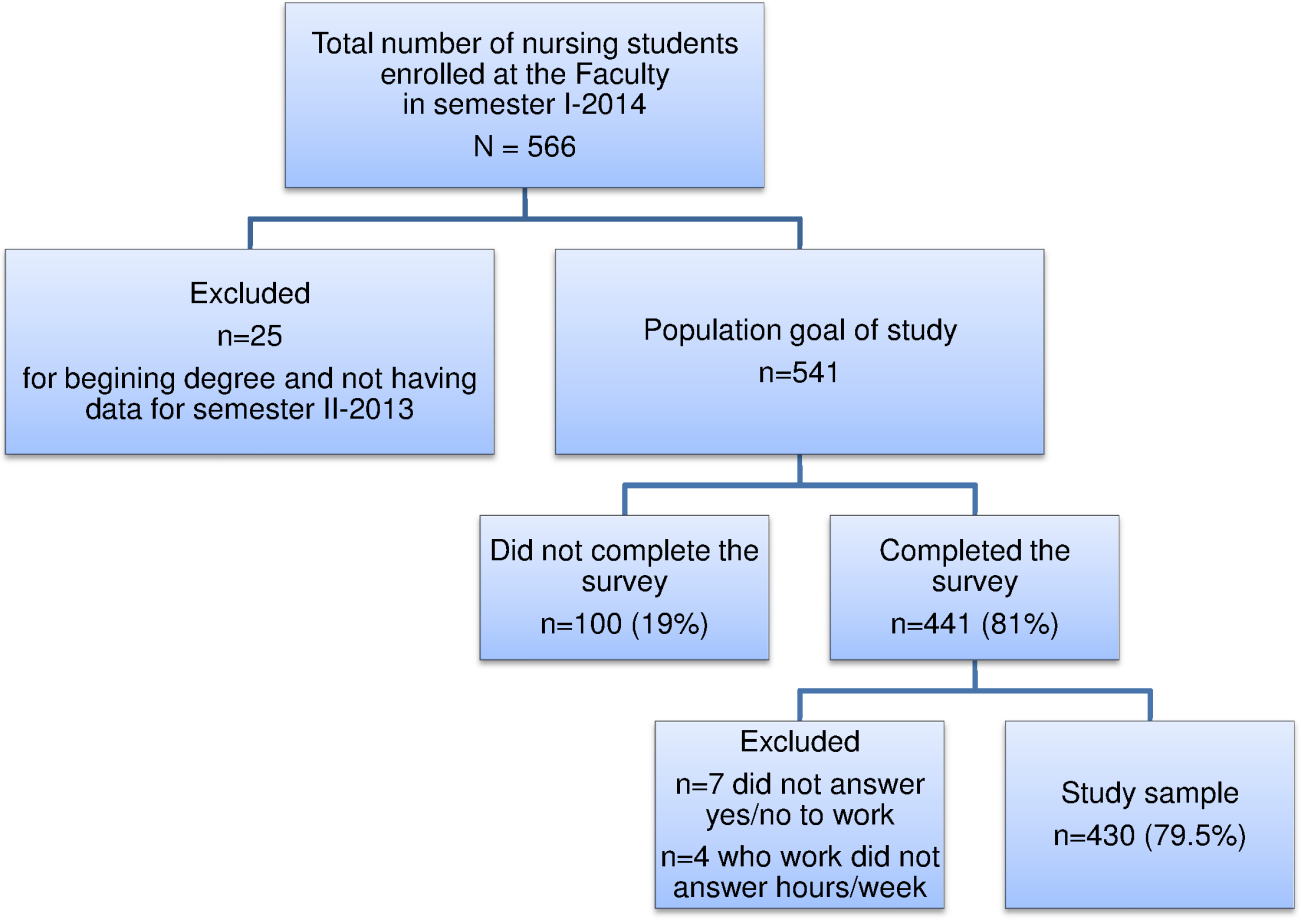
Flow chart of nursing students enrolled at the National University of Bogotá (Colombia) participating in the study.

## Results

Of the 566 students enrolled, 25 who had just started the program were eliminated because of lack of data from the previous semester (first semester, first year students), leaving 541, of whom, 441 participated in the survey (81%). Of these, 11 were excluded for not answering questions about paid work and/or number of hours/week, which left 430 (79.5%) (Fig. 1).

The variables studied were expressed in the Table 1. Of the 430 students, 311 were women (72.3%) and 119 (27.7%) were men. Regarding work, 219 worked during semester II-2013 (50.9%), and 211 did not work (49.1%) (Table 1). The rest of variables studied were expressed in the Table 1.

**Table 1:** Variables of students at the School of Nursing of the National University of Colombia (Bogota) during the second semester of 2013, collected by student survey during the first semester of 2014.

Qualitative variables	Nt = 430, *n*(%)
Sex (female)	311(72.3)
Work (yes)	219(50.9)
Semester in course:	
2	59(13.4)
3	43(9.8)
4	43(9.8)
5	58(13.2)
6	49(11.1)
7	43(9.8)
8	63(14.3)
9	56(12.7)
10	27(6.1)
**Quantitative variables**	**Median (Interval Q3–Q1)**
Age (years)	22(3)
Courses enrolled	4(1)
Courses not enrolled	1(1)
Credits enrolled	16(4)
GPA (Grade point average)	3.8(0.4)
Class Attendance (h/week)	30(2)
Individual study (h/week)	15(10)

By analyzing working hours per week, 150 (54.5%) worked more than 20 h per week and 99 (44.5%) worked less than 20 h per week. The main reasons given for work were socioeconomic 198 (90.4%) and to gain autonomy of the parents and to obtain professional experience (for example working as nursing assistants) 19 (8.7%). Regarding the workplace, 11 (5.0%) worked on the university campus, 144 (66.1%) worked off campus and 63 (28.9%) worked both on and off campus. Most work schedules included both day and night shifts (61.2%) and all the days of the week including Sundays (93.2%). As for the decision to work, 155 (71.4%) preferred to study exclusively, 57 (26.3%) preferred to both work and study and only 3 (1.4%) preferred work exclusively.

The median (interquartile interval) of average grade obtained in the last semester studied was 3.6(5.0) for students in the first semester and 3.7(4.0); 3.7(4.0); 3.9(3.0); 3.8(6.0); 3.7(3.0); 3.9(3.0); 3.8(4.0) y for the ninth semester, 4.0(4.0), with significant differences (Kruskal–Wallis test *p* < 0.001), although a clear increasing or decreasing tendency according the progression of the degree was not observed. Grades for the tenth semester were not collected as these students had graduated and were no longer at the university.

The quantitative variables analyzed regarding the work group are presented in Table 2, which shows that students who work are slightly older, cannot enroll or attend the same number of courses and credits as their colleagues, lose more credits, have lower grades, and take longer to finish their degrees, all of which is significant.

**Table 2:** Variables analyzed (median and interquartile interval) nursing students from the National University of Colombia (Bogota), according to work schedule.

Variables analyzed	Don’t work (*n* = 211)	Work ≤ 20 h/week (*n* = 120)	Work > 20 h/week (*n* = 99)	*P* (Kruskal–Wallis test)
Hours worked (h/week)	0	16(8)	31(4)	<0.001
Age (years)	21(4)	21(3)	23(5)	<0.001
Courses unable to enroll	0(2)	1(2)	2(3)	0.001
Courses studied	4(1)	4(1)	4(1)	0.011
Credits gained	16(5)	17(3)	16(5)	0.008
Credits lost	0(0)	0(0)	0(3)	0.015
Grade point average II-2013	4(5)	5(4)	5(3)	0.001
% Semester delayed	25(50)	25(50)	50(44)	0.003

Regarding the indicators of academic success (Table 3), success rates regarding grades, loss of credits and delay in semesters, it is clear that these success rates diminish significantly as the intensity of paid work increases, except in the case of courses failed with no significant difference.

**Table 3:** Indicators of academic success (number and percentages) according to workgroup.

Indicators of academic success	Don’t work (*N* = 211) *n*(%)	Work ≤ 20 h/week (*N* = 120) *n*(%)	Work > 20 h/week (*N* = 99) *n*(%)	*p* (test *χ*^2^)
Grade point average ≥ 3.0	203(98.1)	117(97.5)	88(89.8)	0.008
Did not lose credits	171(91.4)	96(94.2)	88(84.4)	0.041
Did not fail courses	174(83.1)	97(80.8)	72(72.7)	0.130
Did not delay a semester	87(40.5)	39(32.5)	25(25.3)	0.018

Regarding student participation in extracurricular activities, 71.4% (307) said they did not participate, which distributed by work groups resulted in 71.1% (150) who do not work, 68.3% (82) who work ≤20 h/week and 75.8% who work >20 h/week. The type of activities in which they participated were 14.7% (65) in sports, 4.8% (13) in cultural activities, 2.9% (13) in scientific activities and the remaining a combination of one or more activities. Reasons for not participating, the 253 who responded identified: 37.2% due to time; 9.5% due to study; 9.3% because of work; 9.1% because of lack of interest; and 4.8% because of lack of information. All others gave other reasons.

**Figure 2:**
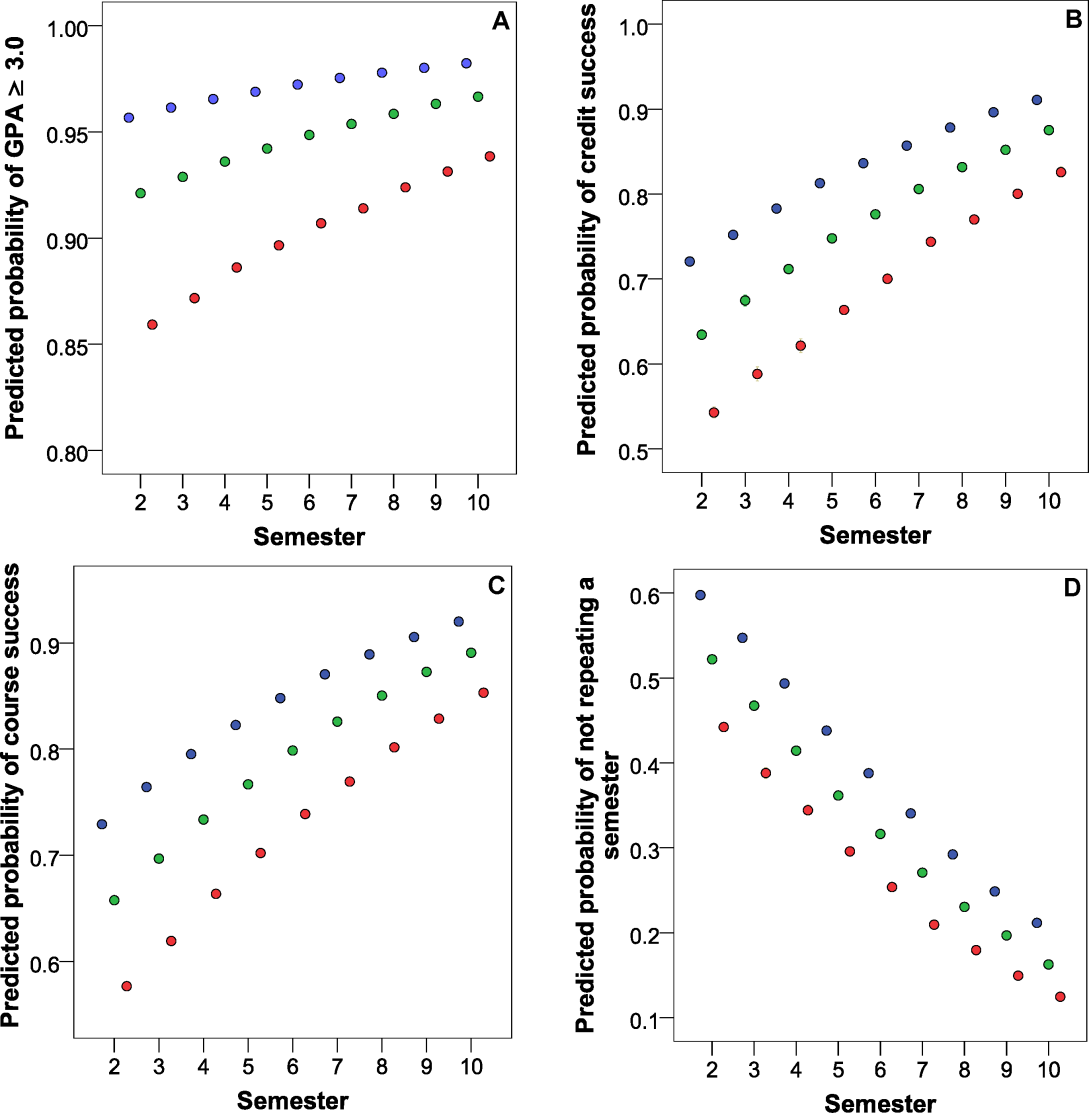
Predicted probability of success according to the grades of the last semester by logistic regression analysis in relation to the semester in course and the type of work among nursing students. No work, blue circles; work ≤ 20 h/week, green circles and work > 20 h/week, red circles. (A) GPA; (B) Credits; (C) Courses; (D) Semesters Notes: CI, confidence interval; GPA, grade point average.

The bivariate logistic regression model was used to analyze the success of each group by weekly working hours (no work, work ≤ 20 h/week and work > 20 h/week), sex and current semester. A significant correlation was established in the work and current semester groups in relation to the four parameters of success in students (GPA ≥ 3, obtaining all credits, all courses and not delaying any semesters. Sex was not an influence; Table 4). Predicted probabilities of obtaining a score of 3.0 or higher, obtaining all credits, all courses and not delaying any semesters, were calculated from this data which is presented in Fig. 2. In the four panels of the figure, the probability of success is shown to be greater in those who do not work, followed by those who work 20 h a week or less. Another important factor is the semester in course as the probability of success increases with each semester completed. Similarly, although it appears contradictory, it is logical that delaying a semester is more likely when working more hours, but also with each semester completed there is a great probability that one of them was repeated.

**Table 4:** Logistic bivariate regression of success in grades (Grade point average ≥ 3.0), credits gained, courses gained and not having delayed semesters in relation to sex, work (no work, work ≤ 20 hs/week and work > 20 h/week) and current semester.

	B(CI 95%) (*vs.* grade point average)	*P*	B(CI 95%) (*vs.* credits)	*P*	B(CI 95%) (*vs.* courses)	*P*	B(CI 95%) (*vs.* on track of semester)	*P*
Sex (female)	1.05(0.41–2.72)	0.921	0.93(0.55–1.56)	0.773	1.09(0.60–1.76)	0.920	1.06(0.66–1.70)	0.808
Work	0.52(0.31–0.89)	0.017	0.69(0.51–0.92)	0.011	0.71(0.53–0.96)	0.024	0.72(0.55–0.95)	0.019
Current semester	1.12(0.94–1.34)	0.202	1.19(1.08–1.31)	<0.001	1.20(1.09–1.33)	<0.001	0.81(0.74–0.88)	<0.001
Model	*χ*^2^ = 6.991; *p* = 0.072		*χ*^2^ = 18.064; *p* < 0.001		*χ*^2^ = 16.837; *p* = 0.001		*χ*^2^ = 34.235; *p* < 0.001

**Notes.**

Work categorized by: no work = 0; work ≤ 20 h/week = 1 and work > 20 h/week = 2. Current semester categorized from 2 to 10.

## Discussion and Conclusions

In Colombia, there is a high percentage (50.3%) of nursing students who work, similar to reported percentages of working university students in Europe 47%; USA 49% and Australia 50% (Beerkens, Mägi & Lill, 2011). This reality regardless of the country is consistent with what has been said by (Guzmán, 2004) that students are increasingly student-workers, ignoring the dichotomy of study or work. The main cause identified was economic (85.5%) which concurred with other studies done in other countries (Beerkens, Mägi & Lill, 2011; Hasson, McKenna & Keeney, 2012). Therefore, if the decision to work is a result of economic necessity, the cause is external to the individual, and is a decision by force majeure. This condition is separated from the theoretically proposed orientation put forward by Warren (Warren, 2002), because students work because of necessity, not by orientation. These facts are confirmed by the high percentage of students who answered that if they could choose between studying and work, they would rather study exclusively (76%). But even accepting that the main cause is economic and is induced by necessity, there are also other reasons which are voluntary (as gaining autonomy and experience), which leads to enrich the theoretical debate surpassing the two apparent dichotomies: to study or to work.

Further research on the possible future impact on higher education and the labor sector in nursing is needed as the relationship between the two sectors is clear regardless of the country, because they use students in patient care as a support to work positions as nursing assistants until they finish their university degree, reconciling both functions.

Another aspect that deserves to be discussed is the number hours worked per week. Our data indicate that 44.5% of student-workers work more than 20 h per week and that their performance suffers, specifically in respect to grades, with delays in completing their degree and failed credits. These findings are consistent with those found in the USA by Moore (Moore, 2008), who notes that working students prefer to prolong their university studies obtaining their degree a few semesters late order to avoid affecting their grades, and those working more than 30 h a week are less likely to graduate.

It is interesting to note that failing courses is not significant, which can be explained by the ability of the student to enroll in a course and then cancel. In many cases this is the cause of loss of credits, but not course, because if they fail a course due to absenteeism or low grades, they risk not reaching a 3.0 average and thereby dropping out of the program altogether. This fact is corroborated by the increase in predicted probability of obtaining a greater than 3.0 grade point average when one works less hours and is further along in the program, which gives the student more experience in choosing which and how many courses to take, without knowing the degree of difficulty in achieving the grade in the credits obtained according to progress of academic studies. However, it is necessary to point out the high predicted risk of failure and delay in finishing among students who work more than 20 h a week, as indicated by previous studies in nursing (Salamonson & Andrew, 2006; Moore, 2008; Rochford, Connolly & Drennan, 2009; Salamonson et al., 2012; Reyes et al., 2012).

As students advance in the degree program they gain an accumulated experience that reduces this difference; this may help to explain that students have learned to reduce the risk and decided to finish their degree later by a few semesters, as shown by the results. Also, this finding can help demonstrate that one of the reasons for the high dropout rate in the first year is because of intense schedules.

The double condition of student-worker is very important in health-related degrees, and especially for nursing, for three reasons: (i) there are students that enroll in the university and work as nursing assistants; working at night and studying during the day; (ii) for integration into the workforce, in jobs related to nursing care, as they advance in their studies; (iii) as it is an experiment-based program, it requires many hours doing internships in hospitals, and according to the Pan-American Health Organization (OMS by its initials in Spanish), nursing is classified as a highly experiment-based program.

Regarding the participation of students in extracurricular university activities (co-curricular), only 29% participated in any of these activities without statistical significance in academic performance, or hours of independent study. Low participation in these activities is unfortunate, because it represents lost opportunities in learning, work readiness, knowledge of global issues, skills, social networks, etc. (Storey, 2010).

There is no solid theoretical body (Riggert et al., 2006), which relates work hours with academic performance (Warren, 2002). One difficulty is that the issue involves several variables, those related to the student, work, type of academic program and context. The main limitation of this research is that it has addressed only some of these dimensions and attempting to cover all of them runs the risk of losing oneself in its immensity. But this very reason helps reinforce the need for specific research in nursing.

Another aspect of discussion are the indicators that show different concepts of academic achievement. Those included in this study are the requirements of the nursing program of the National University of Colombia. Qualitative studies carried out with student-workers are needed in order to obtain contributions to its meaning.

In conclusion, with the data obtained, in the students analyzed and with referred study program, academic performance is affected for nursing students who work more than 20 h per week, especially in the first semesters of the program, compared to students who do not work or work less than 20 h per week; this performance is measured by grade point average, credits lost, and the probability of not finishing the degree in the time and semester established by the program. Sex is not a significant determinant factor.

Further research that addresses these issues is required to further the debate, especially because of the experimental nature of nursing and the socio-economic and cultural differences of each country noted by other authors and collected in the meta-analysis executed by Richardson, Abraham & Bond (2012).

## Supplemental Information

10.7717/peerj.1838/supp-1 Supplemental Information 1 Raw data

## Additional Information and Declarations

### Data Availability

The following information was supplied regarding data availability: The raw data has been supplied as Supplemental Information 1.
